# Prediction of newborn’s body mass index using nationwide multicenter ultrasound data: a machine-learning study

**DOI:** 10.1186/s12884-021-03660-5

**Published:** 2021-03-02

**Authors:** Kwang-Sig Lee, Ho Yeon Kim, Se Jin Lee, Sung Ok Kwon, Sunghun Na, Han Sung Hwang, Mi Hye Park, Ki Hoon Ahn, Korean Society of Ultrasound in Obstetrics and Gynecology Research Group

**Affiliations:** 1 AI Center, Korea University College of Medicine, Seoul, South Korea; 2 Department of Obstetrics and Gynecology, Korea University College of Medicine, 73 Goryeodae-ro, Seongbuk-gu, Seoul, 02841 South Korea; 3 Department of Obstetrics and Gynecology, Kangwon National University Hospital, Kangwon National University School of Medicine, Kangwon, Chuncheon, South Korea; 4 Department of Preventive Medicine, Kangwon National University School of Medicine, Kangwon, Chuncheon, South Korea; 5 Department of Obstetrics and Gynecology, Research Institute of Medical Science, Konkuk University School of Medicine, Seoul, South Korea; 6 Department of Obstetrics and Gynecology, Ewha Medical Institute, Ewha Medical Center, Ewha Womans University College of Medicine, Seoul, South Korea

**Keywords:** Newborn, Body mass index, Estimated fetal weight, Abdominal circumference

## Abstract

**Background:**

This study introduced machine learning approaches to predict newborn’s body mass index (BMI) based on ultrasound measures and maternal/delivery information.

**Methods:**

Data came from 3159 obstetric patients and their newborns enrolled in a multi-center retrospective study. Variable importance, the effect of a variable on model performance, was used for identifying major predictors of newborn’s BMI among ultrasound measures and maternal/delivery information. The ultrasound measures included biparietal diameter (BPD), abdominal circumference (AC) and estimated fetal weight (EFW) taken three times during the week 21 - week 35 of gestational age and once in the week 36 or later.

**Results:**

Based on variable importance from the random forest, major predictors of newborn’s BMI were the first AC and EFW in the week 36 or later, gestational age at delivery, the first AC during the week 21 - the week 35, maternal BMI at delivery, maternal weight at delivery and the first BPD in the week 36 or later. For predicting newborn’s BMI, linear regression (2.0744) and the random forest (2.1610) were better than artificial neural networks with one, two and three hidden layers (150.7100, 154.7198 and 152.5843, respectively) in the mean squared error.

**Conclusions:**

This is the first machine-learning study with 64 clinical and sonographic markers for the prediction of newborns’ BMI. The week 36 or later is the most effective period for taking the ultrasound measures and AC and EFW are the best predictors of newborn’s BMI alongside gestational age at delivery and maternal BMI at delivery.

**Supplementary Information:**

The online version contains supplementary material available at 10.1186/s12884-021-03660-5.

## Background

Low birthweight and childhood obesity are the leading causes of disease burden in the world. One in every seven babies was born with low birthweight (less than 2500 g) in the world for year 2015 and newborns with low birthweight are more likely to die in the first 28 days of life than their normal counterparts [1]. Likewise, 40 million children under the age of five were overweight or obese in the world for year 2016 [2] and childhood overweight or obesity is expected to have short-term and long-term consequences including asthma [3], depression [4], diabetes [5], hypertension [6], dyslipidemia [7] and cardiovascular disorders [8]. Given this global challenge, member states of the World Health Organization endorsed “No Increase in Childhood Overweight by 2025” as one of six global nutrition targets [9].

In this context, several retrospective studies of obstetric patients and their newborns endeavored to analyze newborn’s weight and its major predictors [10–13]. These studies focused on ultrasound measures and maternal/delivery information, while coming from various regions including East Asia (Taiwan), Middle East (Lebanon), North America (United States) and North Europe (Denmark). Based on the linear-regression results of these studies, the following variables were good predictors of newborn’s weight: abdominal circumference or diameter, biparietal diameter, gestational age at delivery, maternal weight at delivery and maternal body mass index (BMI). However, these studies did not address (1) which predictors are more important for the prediction of newborn’s weight and (2) which periods are more effective for taking the ultrasound measures and managing the delivery outcome. Also, existing literature ignores newborn’s BMI and highlights newborn’s weight. However, newborn’s BMI, which has a strong association with newborn’s fat mass, would be a better indicator of newborn’s adiposity, given that newborn’s weight includes not only fat mass but also head size, lean mass and bone mass.

For this reason, this study introduces machine learning approaches to predict newborn’s BMI based on ultrasound measures and maternal/delivery information. Machine learning (or data mining) methods are statistical methods to extract knowledge from large amounts of data. Specifically, the random forest and the artificial neural network (ANN) do not require unrealistic assumptions of linear regression such as ceteris paribus, “all the other variables staying constant”. Also, the random forest can address (1) which predictors are more important for the prediction of newborn’s BMI and (2) which periods are more effective for taking the ultrasound measures and managing the delivery outcome. Indeed, data in this study are larger than those in the previous studies - 4590 mother-baby pairs and 64 independent variables. This study attempts to demonstrate that machine learning approaches based on ultrasound measures would be a useful noninvasive tool for predicting newborn’s BMI.

## Methods

### Participants and variables

Data came from the medical records of 3159 obstetric patients and their newborns enrolled in a multi-center retrospective study. This study was conducted during September 2019–April 2020 and 48 general hospitals participated in this study. This study was approved by the institutional review boards of the general hospitals. This process was followed by data collection, analysis and interpretation. One hundred women with singleton pregnancies were selected from each of the general hospitals. These women were Korean citizens aged 20–44 years. They gave births during June 2015–June 2019 and their gestational age at delivery varied from 24 weeks 0 days to 41 weeks 6 days. These women did not have any disease including pre-gestational or gestational diabetes or hypertension. Newborns who were large for gestational age or had fetal growth restrictions were included, whereas those with congenital anomalies were excluded.

The dependent variable was newborn’s BMI. Newborn’s weight and height were measured at the time of birth and newborn’s BMI was calculated from these measures. The following 64 independent variables were included in this study. Maternal information covered age (years), term births, preterm births, abortions, children alive, height, pre-gestational weight and weight at delivery, and pre-gestational BMI and BMI at delivery. Gestational age (W/D: weeks/days) and two ultrasound measures were taken once during the week 11 - week 13 (GA11): crown-rump length (CRL) (mm) and nuchal translucency (NT) (mm). These indicators were denoted by GA11W1, GA11D1, GA11CRL1 and GA11NT1. Then, gestational age (W/D: weeks/days) and five ultrasound measures were taken once during the week 14 - week 19 (GA14), once in the week 20 (GA20), three times during the week 21 - week 35 (GA21) and once in the week 36 or later (GA36): biparietal diameter (BPD) (mm), head circumference (HC) (mm), abdominal circumference (AC) (mm), femur length (FL) (mm) and estimated fetal weight (EFW) (g). These indicators got the notations of: (1) GA14W1, GA14D1, GA20W1, GA20D1, GA21W1, GA21D1, GA21W2, GA21D2, GA21W3, GA21D3, GA36W1 and GA36D1 (gestational age); (2) GA14BPD1, GA20BPD1, GA21BPD1, GA21BPD2, GA21BPD3 and GA36BPD1 (biparietal diameter); (3) GA14HC1, GA20HC1, GA21HC1, GA21HC2, GA21HC3 and GA36HC1 (head circumference); (4) GA14AC1, GA20AC1, GA21AC1, GA21AC2, GA21AC3 and GA36AC1 (abdominal circumference); (5) GA14FL1, GA20FL1, GA21FL1, GA21FL2, GA21FL3 and GA36FL1 (femur length); and (6) GA14EFW1, GA20EFW1, GA21EFW1, GA21EFW2, GA21EFW3 and GA36EFW1 (estimated fetal weight). For example, GA21BPD1 means the first BPD taken during the week 21 - week 35, whereas GA36EFW1 means the first EFW taken in the week 36 or later. For the calculation of EFW, all general hospitals used the Hadlock’s formula [14] (except one general hospital that used a formula from Shinozuka et al. [15]). These formulas share the same parameters (BPD, AC, FL) and are reported to show similar performances for the prediction of newborn’s weight [16]. Finally, delivery/newborn information covered gestational age at delivery (weeks and days), caesarean delivery methods (no vs. yes), newborn’s sex - female (no vs. yes), Apgar scores in 1 and 5 min after delivery, and neonatal intensive care unit hospitalization (no vs. yes). These variables had missing rates lower than 30% in general and their missing values were replaced by their median values.

### Analysis

Five machine learning methods were applied for predicting newborn’s BMI, the dependent variable of this study: linear regression, random forest and ANNs with one, two and three hidden layers [17]. Each hidden layer had three neurons in this study. Data on 3159 participants were divided into training and validation sets with a 75:25 ratio (2370 vs. 789 observations). The mean squared error (MSE), the average of the squares of errors among 789 observations, was introduced as a criterion for validating the models trained. Here, errors are gaps between actual and predicted values of the dependent variable, newborn’s BMI. Variable importance from the random forest, the effect of a variable on model performance, was used for identifying major predictors of newborn’s BMI among ultrasound measures and maternal/delivery information. R-Studio was employed for the analysis on April 2020.

## Results

Descriptive statistics for continuous and categorical variables in this study are summarized in Table 1. The median (Q2) values of newborn’s BMI, GA36AC1, GA36EFW1 and gestational age at delivery were 12.74 kg/m^2^, 322 mm, 2866 g and 38 weeks, respectively. Likewise, the median values of GA21AC1 and maternal BMI at delivery were 214.70 mm and 26.04 kg/m^2^, respectively. The MSEs of the five machine learning models are shown in Table 2. The random split and the statistical analysis were repeated 3 times and their average MSE was calculated for each of the five statistical methods, i.e., linear regression, random forest and ANNs with one, two and three hidden layers. Linear regression and the random forest were much better models than the ANNs for predicting newborn’s BMI. Their average MSEs over the three runs were 2.0744, 2.1610, 150.7100, 154.7198 and 152.5843, respectively.

**Table 1:** Descriptive Statistics

Continuous Variable	*SD*	*Min*	*Q1*	*Q2*	*Q3*	*Max*
Newborn’s BMI	1.54	6.61	11.95	12.74	13.61	41.10
Maternal Age	4.01	19.00	31.00	33.00	36.00	48.00
Maternal Term Births	0.69	0.00	0.00	0.00	1.00	4.00
Maternal Preterm Births	0.34	0.00	0.00	0.00	0.00	3.00
Maternal Abortions	0.82	0.00	0.00	0.00	1.00	7.00
Children Alive	0.73	0.00	0.00	0.00	1.00	5.00
Maternal Height (cm)	5.17	140.00	158.00	161.00	165.00	181.00
Maternal Pregestational Weight (kg)	8.11	34.00	51.00	55.00	60.00	99.00
Maternal Weight at Delivery Time (kg)	8.60	45.00	62.85	68.00	74.00	92.80
Maternal Pregestational BMI	3.11	14.50	19.49	21.05	23.23	39.86
Maternal BMI at Delivery Time	3.11	16.33	24.21	26.04	28.23	40.00
Number of Ultrasound Equipment Types	2.24	1.00	2.00	2.00	3.00	15.00
GA11W1	0.62	11.00	11.00	11.00	12.00	13.00
GA11D1	1.95	0.00	1.00	3.00	4.00	6.00
GA11CRL1 (mm)	8.66	32.60	50.00	56.00	61.40	79.80
GA11NT1 (mm)	1.19	0.04	1.00	1.20	1.50	40.00
GA14W1	0.86	14.00	15.00	16.00	16.00	19.00
GA14D1	1.91	0.00	1.00	3.00	4.00	6.00
GA14BPD1 (mm)	3.68	23.10	32.40	34.70	36.40	67.00
GA14HC1 (mm)	10.29	72.60	123.70	126.40	128.70	200.00
GA14AC1 (mm)	11.58	34.00	101.40	107.30	112.00	219.00
GA14FL1 (mm)	2.96	9.10	18.00	19.80	21.30	32.50
GA14EFW1 (g)	31.88	14.00	137.00	152.00	165.00	345.00
GA20W1	0.00	20.00	20.00	20.00	20.00	20.00
GA20D1	1.98	0.00	1.00	3.00	5.00	6.00
GA20BPD1 (mm)	4.57	38.00	48.70	51.40	54.00	67.70
GA20HC1 (mm)	14.27	118.40	182.10	191.20	195.60	250.50
GA20AC1 (mm)	42.18	108.70	157.00	166.70	175.00	2113.30
GA20FL1 (mm)	3.67	25.90	32.80	35.00	37.30	45.50
GA20EFW1 (g)	110.31	109.00	367.00	425.00	481.00	980.00
GA21W1	2.18	21.00	24.00	25.00	27.00	35.00
GA21D1	1.97	0.00	1.00	3.00	5.00	6.00
GA21BPD1 (mm)	6.62	46.70	61.45	65.80	70.50	85.00
GA21HC1 (mm)	22.76	169.70	233.50	244.20	249.10	839.00
GA21AC1 (mm)	24.22	108.30	200.50	214.70	231.60	310.50
GA21FL1 (mm)	5.16	32.40	43.90	47.00	51.00	62.40
GA21EFW1 (g)	302.32	177.00	731.00	868.00	1098.00	2185.00
GA21W2	2.10	24.00	28.00	30.00	31.00	35.00
GA21D2	1.90	0.00	1.00	3.00	5.00	6.00
GA21BPD2 (mm)	5.57	61.00	73.60	77.40	80.60	92.90
GA21HC2 (mm)	15.30	193.20	276.90	283.00	284.60	386.20
GA21AC2 (mm)	23.64	155.00	244.00	258.40	272.10	369.70
GA21FL2 (mm)	4.58	43.40	53.40	56.40	59.20	68.60
GA21EFW2 (g)	393.44	644.00	1293.00	1508.00	1747.00	3569.00
GA21W3	1.41	21.00	32.00	33.00	34.00	35.00
GA21D3	1.69	0.00	2.00	3.00	4.00	6.00
GA21BPD3 (mm)	3.71	74.50	82.40	85.00	86.20	93.60
GA21HC3 (mm)	10.46	201.30	306.20	307.00	307.00	390.60
GA21AC3 (mm)	17.49	227.00	280.90	293.00	297.60	381.40
GA21FL3 (mm)	3.10	55.00	61.00	63.20	64.00	69.70
GA21EFW3 (g)	322.77	1211.00	1953.00	2186.00	2273.00	3661.00
GA36W1	0.75	36.00	36.00	36.00	37.00	40.00
GA36D1	1.83	0.00	1.00	3.00	4.00	6.00
GA36BPD1 (mm)	3.29	75.10	89.00	90.80	92.60	103.40
GA36HC1 (mm)	9.36	206.00	323.10	324.70	326.00	419.90
GA36AC1 (mm)	15.32	243.60	314.00	322.00	330.50	460.10
GA36FL1 (mm)	2.79	56.00	67.00	68.90	70.30	89.00
GA36EFW1 (g)	299.59	1577.00	2706.00	2866.00	3036.00	4172.00
Pregnancy Duration - Delivery (Weeks)	1.53	20.00	38.00	38.00	39.00	42.00
Pregnancy Duration - Delivery (Days)	2.00	0.00	1.00	3.00	5.00	6.00
Apgar Score in 1 Minute After Delivery	0.50	0.00	8.00	8.00	9.00	20.00
Apgar Score in 5 Minutes After Delivery	0.50	0.00	9.00	9.00	10.00	10.00
**Categorical Variable**		*No*		*Yes*		*Yes (%)*
Caesarean Delivery		1661		1498		47.42
Newborn’s Sex - Female		1664		1495		47.33
Neonatal Intensive Care Unit Hospitalization		2805		354		11.21

Notes

*SD* Standard Deviation

*AC* Abdominal Circumference (mm)

*BMI* Body Mass Index (kg/m^2^)

*BPD* Biparietal Diameter (mm)

*CRL* Crown-Rump Length (mm)

*EFW* Estimated Fetal Weight (g)

*FL* Femur Length (mm)

*HC* Head Circumference (mm)

*NT* Nuchal Translucency (mm)

*GA11* Gestational Age, Week 11 - Week 13

*GA14* Gestational Age, Week 14 - Week 19

*GA20* Gestational Age, Week 20

*GA21* Gestational Age, Week 21 - Week 35

*GA36* Gestational Age, Week 36 or Later

*W/D* Pregnancy Duration - Weeks/Days

**Table 2:** Model Performance: Mean Squared Error

Model	*Run 1*	*Run 2*	*Run 3*	*Average*
Linear Regression	1.7933	1.9526	2.4774	2.0744
Random Forest	1.8359	2.0782	2.5688	2.1610
Artificial Neural Network 1 Layer	140.0307	158.5399	153.5595	150.7100
Artificial Neural Network 2 Layers	140.0916	158.5026	165.5652	154.7198
Artificial Neural Network 3 Layers	139.3295	158.6813	159.7421	152.5843

**Fig. 1:**
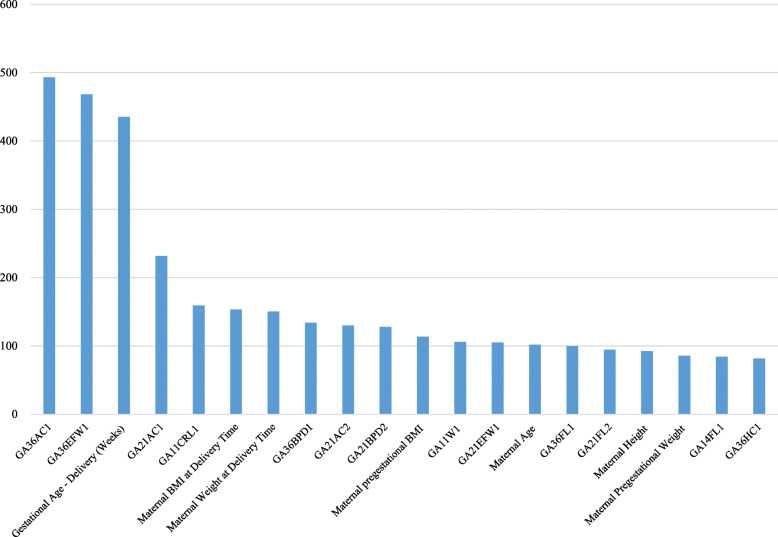
Variable Importance Values of Top 20 Predictors for Newborn’s Body Mass Index from the Random Forest. **Notes***. AC:* Abdominal Circumference. *BMI:* Body Mass Index. *BPD:* Biparietal Diameter. *CRL:* Crown-Rump Length. *EFW:* Estimated Fetal Weight. *FL:* Femur Length. *HC:* Head Circumference. *NT:* Nuchal Translucency. *GA11:* Gestational Age, Week 11 - Week 13. *GA14:* Gestational Age, Week 14 - Week 19. *GA20:* Gestational Age, Week 20. *GA21:* Gestational Age, Week 21 - Week 35. *GA36:* Gestational Age, Week 36 or Later. *W/D:* Gestational Age - Weeks/Days

Based on variable importance from the random forest, major predictors of newborn’s BMI were the first AC and EFW in the week 36 or later, gestational age at delivery, the first AC during the week 21 - the week 35, maternal BMI at delivery, maternal weight at delivery and the first BPD in the week 36 or later (Table 3, Table S1 (supplementary information) and Fig. 1). The findings of linear regression present useful information about the effect of a major determinant on newborn’s BMI. For example, newborn’s BMI will increase by 0.0142 if GA36AC1 increases by 1 mm. Likewise, newborn’s BMI will increase by 0.4142 if gestational age at delivery increases by 1 week. It is to be noted, however, that the results of linear regression are based on an unrealistic assumption of ceteris paribus, “all the other variables staying constant”. For this reason, the coefficients of some predictors were statistically significant in linear regression but their importance rankings were not high from the random forest, a data-driven approach with no such an assumption of “all the other variables staying constant”. In this context, the findings of linear regression are to be considered as just supplementary information to the variable importance from the random forest.

**Table 3:** Random Forest Variable Importance (VI) and Regression Coefficient from Run 1: Top 40 Variables

Variable	Random Forest		Linear Regression	
	*VI Value*	*VI Rank*	*Coefficient*	*P-Value*
GA36AC1	493	1	*0.0142	0.0002
GA36EFW1	468	2	*0.0007	0.0080
Gestational Age - Delivery (Weeks)	435	3	*0.4142	0.0000
GA21AC1	232	4	0.0023	0.6171
GA11CRL1	159	5	0.0003	0.9525
Maternal BMI at Delivery Time	153	6	*-0.1452	0.0023
Maternal Weight at Delivery Time	150	7	*0.0722	0.0001
GA36BPD1	134	8	0.0223	0.0524
GA21AC2	130	9	− 0.0005	0.9060
GA21BPD2	128	10	−0.0004	0.9759
Maternal Pregestational BMI	113	11	*-0.1060	0.0137
GA11W1	106	12	−0.0208	0.7815
GA21EFW1	105	13	*0.0014	0.0055
Maternal Age	102	14	−0.0069	0.3563
GA36FL1	100	15	−0.0042	0.7725
GA21FL2	95	16	−0.0272	0.1342
Maternal Height	92	17	*-0.0706	0.0002
Maternal Pregestational Weight	86	18	*0.0351	0.0468
GA14FL1	84	19	−0.0241	0.2647
GA36HC1	82	20	*-0.0070	0.0289
GA21EFW2	80	21	*0.0012	0.0019
GA20EFW1	79	22	−0.0006	0.4659
GA21AC3	79	23	0.0016	0.7871
GA21HC2	79	24	−0.0009	0.7748
GA11NT1	77	25	−0.0229	0.2652
GA20FL1	74	26	0.0217	0.2701
GA21FL1	72	27	*-0.0513	0.0031
GA20HC1	70	28	0.0015	0.6991
GA21BPD1	68	29	−0.0188	0.1413
GA21EFW3	68	30	0.0008	0.1122
GA14BPD1	67	31	0.0218	0.1921
GA20BPD1	67	32	0.0000	0.9978
GA20AC1	67	33	−0.0001	0.8142
GA21FL3	65	34	0.0033	0.8730
GA14AC1	64	35	−0.0024	0.6350
Apgar Score in 1 Minute After Delivery	58	36	−0.0130	0.7312
GA14EFW1	55	37	−0.0003	0.8853
GA21HC1	55	38	−0.0017	0.3220
GA21D2	53	39	−0.0197	0.2833
GA21BPD3	53	40	−0.0084	0.6104

Notes

*: P-Value < 0.05.

*AC* Abdominal Circumference (mm)

*BMI* Body Mass Index (kg/m^2^)

*BPD* Biparietal Diameter (mm)

*CRL* Crown-Rump Length (mm)

*EFW* Estimated Fetal Weight (g)

*FL* Femur Length (mm)

*HC* Head Circumference (mm)

*ICU* Intensive Care Unit

*NT* Nuchal Translucency (mm)

*GA11* Gestational Age, Week 11 - Week 13

*GA14* Gestational Age, Week 14 - Week 19

*GA20* Gestational Age, Week 20

*GA21* Gestational Age, Week 21 - Week 35

*GA36* Gestational Age, Week 36 or Later

*W/D* Gestational Age - Weeks/Days

## Discussion

### Findings of study

This study introduced machine learning approaches to predict newborn’s BMI based on ultrasound measures and maternal/delivery information. Based on variable importance from the random forest, the week 36 or later is the most effective period for taking the ultrasound measures and AC and EFW are the best predictors of newborn’s BMI alongside gestational age at delivery and maternal BMI at delivery. These results are consistent with existing literature on the topic [18, 19]. In terms of the MSE for predicting newborn’s BMI, linear regression (2.0744) and the random forest (2.1610) were much better models than ANNs with one, two and three hidden layers (150.7100, 154.7198 and 152.5843, respectively). Indeed, the MSEs of linear regression (2.0744) and the random forest (2.1610) were smaller than the variation of newborn’s BMI (2.4649). This suggests that machine learning approaches based on ultrasound measures would be a useful noninvasive tool for predicting newborn’s BMI.

The findings of this study are consistent with those of previous retrospective studies on the prediction of newborn’s weight with clinical and sonographic markers. In a study of 238 obstetric patients in Denmark, AC and BPD during the third trimester were effective predictors of newborn’s weight, given that the MSE of linear regression was similar with the variation of newborn’s weight [10]. In a study of 109 pregnant women in the United States, newborn’s weight had positive associations with fetal adiposity in the week 30 and gestational age at delivery [11]. In a study of 1000 obstetric patients in Lebanon, newborns with maternal gestational weight gain were more likely to have macrosomia than those with normal gestational weight gain (Odds Ratio 1.888) [12]. Likewise, another study of 110 pregnant women in Taiwan reported that AC and BPD during the week 20 - week 24 are significant predictors of newborn’s weight together with gestational age at delivery, maternal weight at delivery and maternal BMI at delivery [13]. However, the previous studies did not address (1) which predictors are more important for the prediction of delivery outcome and (2) which periods are more effective for taking ultrasound measures and managing delivery outcome. This study provides plausible answers to these challenging questions.

Moreover, conventional studies focus on newborn’s weight as a measure of newborn’s adiposity but the findings of this study suggest that newborn’s BMI would be a good alternative. Firstly, the United States Center for Disease Control and Prevention recommends the BMI-for-age chart as a screening tool for the overweight and underweight of boys and girls aged 2 to 20 years [20]. Two major rationales behind this recommendation state that (1) the BMI is a more consistent indicator across different generations than weight and (2) the BMI contains the dimensions (and strengths) of weight and height measures at the same time. Secondly, it is reported that newborn’s BMI has stronger correlations with magnetic-resonance-imaging measures of newborn’s fat mass than do newborn’s other anthropometrics [21]. Thirdly, infant’s BMI is expected to have a stronger correlation with early childhood obesity than infant’s weight-for-length. Based on the medical records of 73,949 full-term infants from a large pediatric network, 47% of infants with BMI ≥ 97.7th percentile at 2 months (vs. 29% of infants with weight-for-length ≥ 97.7th percentile at 2 months) were obese at 2 years [22]. Fourthly, using newborn’s BMI (instead of newborn’s weight) would engender greater stability for statistical analysis. For example, the estimations of ANNs with two layers did not converge when newborn’s weight (instead of newborn’s BMI) was the dependent variable in this study.

### Limitations of study

This study had some limitations. Firstly, for the calculation of EFW, one general hospital used a different formula. Using the same formula for EFW is expected to improve model performance in future study. However, the results of this study did not change after removing the data based on the different formula. Secondly, this study did not consider possible mediating effects among variables. Thirdly, it would be a good topic for future research to develop a BMI guideline for newborn’s adiposity. According to an international guideline, adult’s categories of underweight, normal, overweight and obesity are defined as BMIs smaller than 18.5 kg/m^2^, within 18.5–25.0 kg/m^2^, within 25.0–30.0 kg/m^2^ and equal to/greater than 30.0 kg/m^2^, respectively [23]. An equivalent guideline for newborns needs to be developed based on comprehensive and systematic analysis. Fourthly, this study did not consider socioeconomic factors (education, income) and other possible obstetric variables such as periodontitis, upper gastrointestinal tract symptoms, gastroesophageal reflux disease, Helicobacter pylori, pelvic inflammatory disease history, diabetes mellitus (type I, type II, gestational), hypertension (chronic, gestational) and medication history (e.g., progesterone, calcium channel blocker, nitrate, tricyclic antidepressant, benzodiazepine and sleeping pills). Recent studies on preterm birth reported that these factors would affect the delivery outcome [24, 25] and it would be an important contribution to extend this study based on these new variables. Fifthly, further analysis of specific patients, e.g., symptomatic vs. asymptomatic, single vs. multiple gestation, would offer more insight on this line of research with more detailed clinical implications. Sixthly, this study did not consider various options of parameter tuning for the ANN. Its performance was worse than those of linear regression and the random forest in this study. Finding optimal parameters for the ANN is reported to be a challenging task and it will be a good topic for future research. Seventhly, the focus of this study was to find important predictors of newborn’s BMI. Exploring possible mechanisms between each important predictor and newborn’s BMI is expected to make a good contribution for this line of research. Finally, the values of the following variables outside 1.5*(Interquartile Range), so called “outliers”, were deleted in this study: maternal weight at delivery, GA11CRL1, GA20BPD1, GA20FL1, GA21BPD1, GA21FL1, GA21BPD2, GA21FL2, GA21BPD3 and GA21FL3. It was beyond the scope of this study to evaluate other optimal strategies to handle outliers in the data.

## Conclusions of study

The week 36 or later is the most effective period for taking the ultrasound measures and AC and EFW are the best predictors of newborn’s BMI alongside gestational age at delivery and maternal BMI at delivery. Machine learning approaches based on ultrasound measures would be a useful noninvasive tool for predicting newborn’s BMI.

## Supplementary Information

**Additional file 1: Table S1** Random Forest Variable Importance (VI) and Regression Coefficient from Run 1: All Variables.

## Data Availability

The datasets used and/or analysed during the current study are available from the corresponding authors on reasonable request.

## Abbreviations

*AC*: Abdominal circumference
*ANN*: Artificial neural network
*BMI*: Body mass index
*BPD*: Biparietal diameter
*CRL*: Crown-rump length
*EFW*: Estimated fetal weight
*FL*: Femur length
*HC*: Head circumference
*IRB*: Institutional review board
*MSE*: Mean squared error
*NT*: Nuchal translucency
*GA11*: Gestational age, week 11 - week 13
*GA14*: Gestational age, week 14 - week 19
*GA20*: Gestational age, week 20
*GA21*: Gestational age, week 21 - week 35
*GA36*: Gestational age, week 36 or later
*W/D*: Gestational age - weeks/days
