# Supplementary material for: Prediction of newborn’s body mass index using nationwide multicenter ultrasound data: a machine-learning study
Source: BMC Pregnancy Childbirth. 2021 Mar 2;21:172. doi: 10.1186/s12884-021-03660-5 (PMC7927215; doi:10.1186/s12884-021-03660-5)
Supplement: Supplementary file 1 — Additional file 1: Table S1 Random Forest Variable Importance (VI) and Regression Coefficient from Run 1: All Variables. [file 12884_2021_3660_MOESM1_ESM.doc]

**Table S1** Random Forest Variable Importance (VI) and Regression Coefficient from Run 1: All Variables

| **Variable** | **Random Forest** | | **Linear Regression** | |
| --- | --- | --- | --- | --- |
|  | *VI Value* | *VI Rank* | *Coefficient* | *P-Value* |
| GA36AC1 | 493 | 1 | *0.0142 | 0.0002 |
| GA36EFW1 | 468 | 2 | *0.0007 | 0.0080 |
| Gestational Age - Delivery (Weeks) | 435 | 3 | *0.4142 | 0.0000 |
| GA21AC1 | 232 | 4 | 0.0023 | 0.6171 |
| GA11CRL1 | 159 | 5 | 0.0003 | 0.9525 |
| Maternal BMI at Delivery Time | 153 | 6 | *-0.1452 | 0.0023 |
| Maternal Weight at Delivery Time | 150 | 7 | *0.0722 | 0.0001 |
| GA36BPD1 | 134 | 8 | 0.0223 | 0.0524 |
| GA21AC2 | 130 | 9 | -0.0005 | 0.9060 |
| GA21BPD2 | 128 | 10 | -0.0004 | 0.9759 |
| Maternal pregestational BMI | 113 | 11 | *-0.1060 | 0.0137 |
| GA11W1 | 106 | 12 | -0.0208 | 0.7815 |
| GA21EFW1 | 105 | 13 | *0.0014 | 0.0055 |
| Maternal Age | 102 | 14 | -0.0069 | 0.3563 |
| GA36FL1 | 100 | 15 | -0.0042 | 0.7725 |
| GA21FL2 | 95 | 16 | -0.0272 | 0.1342 |
| Maternal Height | 92 | 17 | *-0.0706 | 0.0002 |
| Maternal Pregestational Weight | 86 | 18 | *0.0351 | 0.0468 |
| GA14FL1 | 84 | 19 | -0.0241 | 0.2647 |
| GA36HC1 | 82 | 20 | *-0.0070 | 0.0289 |
| GA21EFW2 | 80 | 21 | *0.0012 | 0.0019 |
| GA20EFW1 | 79 | 22 | -0.0006 | 0.4659 |
| GA21AC3 | 79 | 23 | 0.0016 | 0.7871 |
| GA21HC2 | 79 | 24 | -0.0009 | 0.7748 |
| GA11NT1 | 77 | 25 | -0.0229 | 0.2652 |
| GA20FL1 | 74 | 26 | 0.0217 | 0.2701 |
| GA21FL1 | 72 | 27 | *-0.0513 | 0.0031 |
| GA20HC1 | 70 | 28 | 0.0015 | 0.6991 |
| GA21BPD1 | 68 | 29 | -0.0188 | 0.1413 |
| GA21EFW3 | 68 | 30 | 0.0008 | 0.1122 |
| GA14BPD1 | 67 | 31 | 0.0218 | 0.1921 |
| GA20BPD1 | 67 | 32 | 0.0000 | 0.9978 |
| GA20AC1 | 67 | 33 | -0.0001 | 0.8142 |
| GA21FL3 | 65 | 34 | 0.0033 | 0.8730 |
| GA14AC1 | 64 | 35 | -0.0024 | 0.6350 |
| Apgar Score in 1 Minute After Delivery | 58 | 36 | -0.0130 | 0.7312 |
| GA14EFW1 | 55 | 37 | -0.0003 | 0.8853 |
| GA21HC1 | 55 | 38 | -0.0017 | 0.3220 |
| GA21D2 | 53 | 39 | -0.0197 | 0.2833 |
| GA21BPD3 | 53 | 40 | -0.0084 | 0.6104 |
| GA14HC1 | 48 | 41 | 0.0005 | 0.9104 |
| GA21HC3 | 43 | 42 | 0.0061 | 0.1171 |
| GA11D1 | 39 | 43 | -0.0068 | 0.7176 |
| GA14D1 | 39 | 44 | 0.0229 | 0.2386 |
| GA20D1 | 39 | 45 | 0.0021 | 0.8950 |
| GA21W2 | 38 | 46 | *-0.1288 | 0.0024 |
| GA21D1 | 36 | 47 | -0.0082 | 0.6457 |
| GA21D3 | 36 | 48 | -0.0136 | 0.4870 |
| Pregnancy Length - Delivery (Days) | 35 | 49 | *0.0488 | 0.0006 |
| GA36D1 | 33 | 50 | -0.0264 | 0.1125 |
| GA21W1 | 33 | 51 | 0.0013 | 0.9724 |
| GA21W3 | 33 | 52 | *-0.2078 | 0.0000 |
| GA36W1 | 31 | 53 | *-0.1926 | 0.0000 |
| GA20W1 | 31 | 54 | *-0.0927 | 0.0633 |
| Apgar Score in 5 Minutes After Delivery | 24 | 55 | 0.0440 | 0.4425 |
| Maternal Abortions | 23 | 56 | 0.0377 | 0.2817 |
| Number of Ultrasound Equipment Types | 22 | 57 | 0.0119 | 0.3639 |
| Children Alive | 21 | 58 | 0.1141 | 0.2243 |
| Caesarean Delivery Method | 21 | 59 | *0.2956 | 0.0000 |
| Neonatal ICU Hospitalization | 21 | 60 | -0.0065 | 0.9461 |
| Maternal Term Births | 21 | 61 | -0.0327 | 0.7372 |
| GA14W1 | 17 | 62 | 0.0518 | 0.4895 |
| Newborn's Sex - Female | 16 | 63 | *-0.1642 | 0.0046 |
| Maternal Preterm Births | 13 | 64 | -0.0012 | 0.9907 |

**Notes**

| **:* | P-Value < 0.05 |
| --- | --- |
| *AC:* | Abdominal Circumference (mm) |
| *BMI:* | Body Mass Index (kg/m^2^) |
| *BPD:* | Biparietal Diameter (mm) |
| *CRL:* | Crown-Rump Length (mm) |
| *EFW:* | Estimated Fetal Weight (g) |
| *FL:* | Femur Length (mm) |
| *HC:* | Head Circumference (mm) |
| *ICU:* | Intensive Care Unit |
| *NT:* | Nuchal Translucency (mm) |
| *GA11:* | Gestational Age, Week 11 - Week 13 |
| *GA14:* | Gestational Age, Week 14 - Week 19 |
| *GA20:* | Gestational Age, Week 20 |
| *GA21:* | Gestational Age, Week 21 - Week 35 |
| *GA36:* | Gestational Age, Week 36 or Later |
| *W/D:* | Gestational Age - Weeks/Days |
